# Cellular Modifications in Spermatogenesis during Seasonal Testicular Regression: An Update Review in Mammals

**DOI:** 10.3390/ani12131605

**Published:** 2022-06-22

**Authors:** Ester Beltrán-Frutos, Vicente Seco-Rovira, Jesús Martínez-Hernández, Concepción Ferrer, María Isabel Serrano-Sánchez, Luis Miguel Pastor

**Affiliations:** Department of Cell Biology and Histology, Medical School, IMIB-Arrixaca, Regional Campus of International Excellence “Campus Mare Nostrum”, University of Murcia, 30120 Murcia, Spain; ebf96527@um.es (E.B.-F.); vicente.seco@um.es (V.S.-R.); jesus.martinez7@um.es (J.M.-H.); mcferrer@um.es (C.F.); maribel.ss88@gmail.com (M.I.S.-S.)

**Keywords:** proliferation, apoptosis, Sertoli cell, short photoperiod, testes, seminiferous epithelium, seasonal testicular regression

## Abstract

**Simple Summary:**

The most common form of reproduction in mammals is seasonal reproduction. This ensures that offspring are born at the most suitable time for survival, due to the abundance of food and the optimal temperatures for early postnatal development. In males, one way to achieve this is to decrease or lose fertility over a given period. This loss is associated with a greater or lesser degree of spermatogenesis modification that affects both germ and Sertoli cells. This paper reviews the different cellular mechanisms that have been postulated in recent years to explain how the activity of the seminiferous epithelium decreases during the non-reproductive period.

**Abstract:**

Testicular regression occurs during the non-breeding season in many mammals. This affects spermatogenesis, resulting in decreased or arrested activity. Both lead to a decrease or cessation in sperm production. In recent years, the cellular mechanisms that lead to infertility in males in non-reproductive periods have been studied in very different species of mammals. At the start of the present century, the main mechanism involved was considered as an increase in the apoptotic activity of germ cells during the regression period. The loss of spermatogonia and spermatocytes causes not only a decrease in spermatogenesis, but an arrest of the seminiferous epithelium activity at the end of regression. Recently, in some mammal species, it was found that apoptosis is the usual mechanism involved in epithelium activity arrest, although it is firstly atrophied by massive desquamation of the germ cells that are released from their binding with the Sertoli cells, and which are shed into the lumen of the seminiferous tubule. In other species, it has been shown that not only germ cell apoptosis, but also Sertoli cell apoptosis, including decreased proliferative activity, spermatophagy or autophagy, are involved in testicular regression. Furthermore, the most recent studies indicate that there are multiple patterns of seminiferous epithelium regression in seasonally breeding animals, which may not only be used by different species, but also by the same ones to reproduce in the best conditions, ensuring their survival. In conclusion, at this time, it is not possible to consider the existence of a paradigmatic cellular mechanism in the involution of the seminiferous epithelium applicable to all male mammals with seasonal reproduction, rather the existence of several mechanisms which participate to a greater or lesser extent in each of the species that have been studied to date.

## 1. Introduction

In nature, there are animal species that drastically reduce their reproductive capacity at a certain time of the year. This phenomenon is known as seasonal reproduction and is considered a natural mechanism to activate and deactivate the reproductive capacity in adult mammals, which allows for offspring that are born in optimal environmental conditions for their survival [1]. Day length is one of the most important factors regulating seasonal reproduction in these animals. On this basis, animals can be classified into long-day and short-day breeders [2]. During the non-breeding season (in both long-day and short-day breeders), it has been observed that the loss of male fertility is accompanied, in many cases, by a decrease in testicular weight and seminiferous tubule diameter [3].

Different studies have pointed to the existence of diverse cellular changes that are related to the decrease or arrest of spermatogenesis during the non-reproductive season, causing testicular regression or atrophy. One of these changes consists of a reduction in spermatogenesis itself, preserving all germ cell lines, as occurs in ram [4,5], deer [6], foal [7] or naked mole-rat [8]. Another is the arrest or blockage of spermatogenesis without the formation of sperm. For example, this occurs in roe deer [9], black bear [10], red vole [11], prairie dog [12], squirrel [13], hare [14], and the Syrian hamster [15]. Finally, spermatogenesis may be arrested in a state in which the seminiferous tubules are only formed by spermatogonia and Sertoli cells, as occurs with the large hairy armadillo [16], mink [17,18] or bat, yellowish myotis [19], and also in birds, such as ostrich [20] or jungle crow [21]. Evidently, the decrease in spermatogenesis or its arrest is a very important phenomenon since it causes infertility or temporary sterility in males, preventing the reproduction of the species at times of the year that are not favorable for the survival of the offspring.

In 2001, Young and Nelson [22] performed a first review of the different cellular mechanisms involved in spermatogenesis and formulated the hypothesis that the rate of apoptosis increases during testicular regression, whereas little testicular apoptosis is observed during testicular recrudescence or during the breeding season. The authors proposed that the Fas system is involved as a molecular pathway in the apoptosis of germ cells during regression and pointed to differences between rodents and birds in the types of cells that undergo apoptosis, while different cellular mechanisms are involved in testicular regression. Therefore, in birds, such as the European starling (*Sturnus vulgaris*), apoptotic activity would only occur in Sertoli and spermatocyte cells, but only in spermatocyte cells in mammals (for example, in white-footed mouse). Subsequently, in the two decades following the publication of this work, many species have been studied and it has been observed that the conclusions suggested by Young and Nelson [22] should be modified. Indeed, in recent years, several authors have postulated a paradigm shift regarding the suggestions of Young and Nelson [22] without presenting any new or systematical proposal on this issue.

Consequently, the main objective of this review is to summarize and systematize the findings of the last 20 years regarding the cellular mechanisms that cause the regression of the seminiferous epithelium during the non-breeding season of mammals with seasonal reproduction. A second objective is to present the partial explanations that have been proposed on how these mechanisms act in the regression of the seminiferous epithelium. Finally, the third objective is to integrate the many different explanations concerning these cellular mechanisms to facilitate future studies in this important field of reproductive biology.

## 2. Results

### 2.1. Proliferation and Apoptosis in Seminiferous Epithelium

For many years, it has been known that for the process of spermatogenesis to occur correctly, it requires a balance between the proliferation and differentiation of spermatogonia and death by apoptosis of various germ cells [23,24]. Spermatogonia arise from the division of spermatogonia stem cells (SSCs), which are undifferentiated, scarce, and defined by their ability to maintain a balance between self-renewal and differentiation into spermatogonia. This balance is essential to maintain a SSCs pool and respond to the need for more spermatogonia, while maintaining the normal testicular function [25]. For spermatogenesis to develop correctly, the proper functioning of Sertoli cells is essential. These are somatic cells, normally with a columnar and polarized morphology, whose functions include supporting the germ cells by supplying nutrients, acting as a mediator of the external signals necessary for the maintenance of spermatogenesis [26], and playing an essential role in regulating the differentiation of these germ cells [27]. Another essential function of Sertoli cells in the seminiferous epithelium is to act as a phagocytic cell to eliminate any remains of the spermatids and apoptotic germ cells that appear during spermatogenesis [23,28]. 

Apoptosis is a process of programmed cell death, which is associated with morphological changes, such as cell volume reduction, chromatin condensation and margination, vesicle formation in the plasmatic membrane, and the formation of so-called apoptotic bodies. Another feature associated with this process is the rapid elimination of dead cells by cells with a phagocytic capacity before the contents of the dead cells can be released, which would cause an inflammatory reaction [29]. In many mammals, such as rats [30,31], hamsters [32], and humans [33], during the normal process of spermatogenesis, the spontaneous death of germ cells through apoptosis has been observed in many stages of their development. Spontaneous apoptosis of spermatogonia and spermatocytes during meiotic division has been observed in both rats and Syrian hamsters. In mouse, the apoptosis of spermatocytes is observed more frequently, less frequently in spermatogonia, and very rarely in spermatids [29].

A typical characteristic of the Sertoli cell, along with others in the mammal body, is that it is a fully differentiated and quiescent cell [34]. In several animal species, including humans [35] and rats [36], their number does not vary during adulthood, proliferating only during postnatal development [37,38] until they reach a number that remains constant during the rest of adult life. In pathological situations that result in the deterioration of the seminiferous epithelium, Sertoli cells remain constant in number, although they suffer substantial cytoplasmic alterations [39,40]. However, it is currently considered that this cell is not always quiescent in adult mammals, since a turnover of these cells that is mediated by proliferation and apoptosis has been noted [41].

The importance of the balance between the phenomena of proliferation and apoptosis in preserving the function of the seminiferous epithelium has led several authors to investigate this balance in relation to changes in the fertility of males with seasonal reproduction.

### 2.2. Proliferation and Apoptosis in Germinal Cells during the Regression of Seminiferous Epithelium in Seasonally Breeding Mammals

In the testicular regression of males with seasonal reproduction, the data regarding proliferation and apoptosis have been somewhat uneven, but point to their involvement in a wide variety of mammals. For instance, in species whose reproductive period coincides with seasons with short days (short photoperiod) and in which females have a long gestation period, only a decrease in the proliferation of spermatogonia during the non-reproductive season (long photoperiod) has been observed, with apoptosis not playing an important role in the regulation of spermatogenesis. In fact, this reduction in proliferation would be sufficient to generate a decrease in sperm production, as reflected by the presence of a significant increase in the histological sections of the seminiferous tubules with hypospermatogenesis. For example, this method of modifying spermatogenesis may occur in species that have a long gestation period involving fertilization in autumn and the birth of offspring in spring, a time with greater feeding possibilities for the young. This is the case of the roe deer, in which it has been shown that apoptotic activity is not determinant in the slight testicular regression observed in the non-breeding season [42]. Rather, it is the proliferative activity of the spermatogonia that is decreased, indicating that the involution of the seminiferous epithelium is caused by this activity. As we have indicated, the roe deer is a species in which mild testicular regression is very different from what is found in some rodents, where testicular regression due to the short photoperiod implies the non-formation of sperm by the seminiferous epithelium [15].

**Figure 1:**
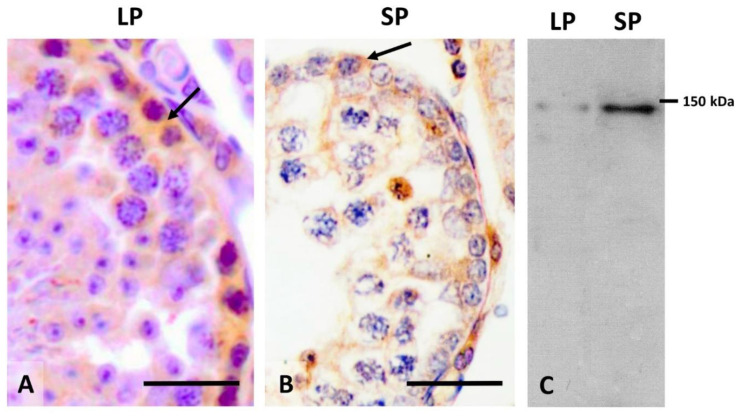
Expression of c-kit in hamster testis in animals subjected to long (LP) and short photoperiod (SP). In (**A**,**B**), positivity is observed in some spermatogonia (arrows) of the seminiferous epithelium in both the normal and fully regressed seminiferous epithelium. In (**C**), strong expression of c-kit in the regressed testes can be clearly observed by Western blotting. Scale bar = 25 µm.

Regarding species that have a short gestation period, they usually undergo spring fertilization and a similar development stage, which allows the offspring to have favorable conditions for feeding, indicating that no sperm are formed as a consequence of the decrease in spermatogenesis. Additional information is available for long gestational species concerning the process of testicular regression than for long gestational species, in which the role of cell proliferation has been extensively studied. Therefore, in studies of Syrian hamsters subjected to a short photoperiod, a decrease in the number of differentiated spermatogonia and an increase in the number of undifferentiated spermatogonia in regressed testes have been described. The spermatogonia probably show an increase in proliferation activity prior to recrudescence to restore the seminiferous epithelium [43]. In other species exposed to a short photoperiod, similar changes have been found during the non-breeding season [43]. A “feedback” mechanism between undifferentiated and differentiated spermatogonia may serve as a regulatory system for spermatogenesis in seasonal breeding animals [43]. Therefore, during testicular regression in Syrian hamsters, it has been found that the proliferation index of spermatogonia is significantly lower during initial regression, recovering gradually during the total regression period, when values similar to those of animals subjected to a long photoperiod are reached [15]. On the one hand, this result suggests that a decrease in spermatogonia would participate in the atrophy of the seminiferous epithelium in the initial testicular regression. On the other hand, the increase observed in the final stage of regression would allow for the restoration of spermatogenesis in the stage of recrudescence that follows [15]. However, in relation to the regulation of proliferation in the seminiferous epithelium during seasonal testicular regression, few studies have been conducted. A widely studied receptor involved in the proliferation of spermatogonia during spermatogenesis is the c-kit (or CD117; stem cell factor) receptor. In hamsters subjected to a short photoperiod, an increase in c-kit receptor expression has been observed after total regression compared with testes in long photoperiod (Figure 1), while expression is further increased at the onset of recrudescence [44]. The role of this receptor has been studied in the seasonal breeding of roe deer, in which it has been found that its expression increases during winter and before the onset of recrudescence [45]. Moreover, it has been observed that in the pre-reproductive period of *Rana esculenta*, there is an increase in c-kit receptor expression in the testes [46]. These findings would support the fact that there is a stage of preparation for recrudescence in the population of spermatogonia, in the sense that an increase in the expression of c-kit would stimulate the proliferation of differentiating type A spermatogonia, and the transition from type B spermatogonia to pre-leptotene and spermatocytes [47].

In relation to apoptosis in the atrophic testes of Djungarian hamsters subjected to a short photoperiod, a marked increase in apoptotic germ cells has been described, with an increase in fragmentation of DNA in the testis, while exposure to a long photoperiod was observed to suppress apoptosis [48]. On the basis of these data, the authors suggested that germ cell apoptosis is one of the earliest events associated with testicular regression induced after exposure to a short photoperiod, while the growth observed in the photo stimulated testis would be associated with a rapid cessation of the apoptotic process [48]. In another study, where the “in situ” apoptosis detection technique (TUNEL) was used in mouse, it was shown that testicular regression induced by a short photoperiod was mediated, at least in part, by the process of apoptosis. The incidence of apoptosis in white-footed mouse, increased more than 3-fold after 6, 8, and 10 weeks of exposure to the short photoperiod compared with control animals [22]. The apoptotic cells were identified as predominantly spermatocytes, although spermatogonia and spermatids were also affected to a lesser extent [22,49,50]. During the period of recrudescence, apoptosis decreased to normal levels [51,52]. Furthermore, an increase in apoptosis delayed testicular recrudescence, but there was no change in proliferative activity in hamsters exposed to a short photoperiod and low temperatures [53]. Recently, in another rodent, it was also found that spermatogenesis during the seasonal reproductive cycle is controlled by proliferation and apoptosis of germ cells, including spermatogonia and primary spermatocytes [54].

In addition to these species, the histology of the testes of several rodents during the non-reproductive period has recently been described: The wood mouse, *Apodemus sylvaticus*, and the Algerian mouse, *Mus spretus*, in syntropic populations of the south of the Iberian Peninsula [55]. Wood mice do not breed in summer and Algerian mice do not breed in winter. Regarding histology, the testes of the wood mice in their non-reproductive period show an arrest in spermatogenesis, which is related to changes in cell adhesion molecules. At that time, serum testosterone levels are decreased. In contrast, the histological pattern of Algerian mice is different in the non-breeding season. On the one hand, involutional changes of the seminiferous epithelium are not observed, even though there is a decrease in serum testosterone levels in the animals. The fact that the seasonal mode of reproduction is significantly different between northern and southern populations of mice, both Algerian and wood mouse, implies that there must be very specific environmental cues or differences between these populations. Similarly, these results show that there may be different cellular mechanisms for the regression of the seminiferous epithelium in the same species, and that one or the other is selected in accordance with certain biological conditions. It is likely that the regression of the seminiferous epithelium in the Algerian mouse depends on a decrease in proliferation. However, in the wood mouse, there is an apoptotic deletion of primary spermatocytes in the regressing testes. The authors believe that apoptosis does not contribute significantly to the loss of germ cells in the seminiferous epithelium during testicular regression. They consider that this apoptosis is only the cause of the epithelium that remains arrested after suffering regression through the desquamation of the germ cells. However, only by continuously studying the regression period would it be possible to know if this hypothesis is true [55]. In contrast, a study of apoptosis, proliferation, and seasonal body changes in various organs in male bank voles (*Myodes glareolus*) found that there were changes in the number of testicular cells in the testes due to an increase in apoptotic cells in the seminiferous epithelium during testicular regression [56]. Another recent study in the same species has identified senescence as a regulator of spermatogenesis in regressed testes. Interactions of low androgen with Irt-like protein 9 (ZIP9), extracellular signal-activated kinase 1/2 (ERK1/2), and cAMP result in germ cell senescence in regressed testes [57]. In addition, a recent report has described how Mediterranean pine vole, *Microtus duodecimcostatus* reproduction depends on cues, such as food or water availability. A male vole captured in wastelands underwent seasonal testis regression in summer, when the seminiferous epithelium only had Sertoli and spermatogonia cells, whereas those living close to poplar plantations or in animal houses reproduced throughout the year. These facts demonstrate that the microenvironment of a particular vole subpopulation could be determinant for the type of reproductive status. It is proposed that these animals are purely opportunistic and photoperiod-independent breeders. Moreover, in this paper, several molecular pathways, including MAPK, were shown to be deregulated and it was considered that during testicular regression there is an “immune privilege” in the testis. The authors suggest that in this species there is a link between this immunological state and the seasonal reduction in testosterone and testis regression, mediated by the deregulation of cell–cell adhesion molecules and disruption of the blood-testis barrier (BTB) dynamics in regressed testis [58].

**Figure 2:**
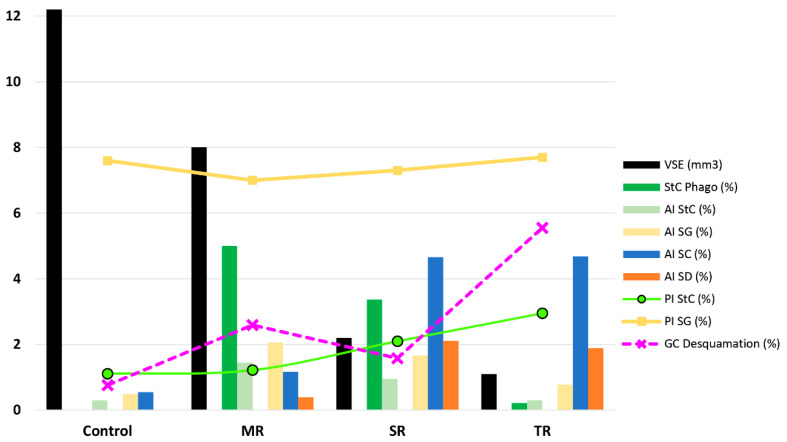
Graph summarizing the results obtained during testicular regression in Syrian hamsters subjected to a short photoperiod. To facilitate the joint representation, some modifications have been conducted to the scale of the studied parameters. **VSE**: Total volume of seminiferous epithelium (mm^3^); **StC Phago (%)**: Percentage of Sertoli cells showing phagocytosis of elongated spermatids; **AI StC (%)**: Sertoli cells apoptotic activity index; **AI Sg (%)**: Apoptotic activity index of spermatogonia; **AI SC (%)**: Spermatocyte apoptotic activity index; **AI SD (%)**: Apoptotic activity index of round spermatids; **PI StC (%)**: Sertoli cells proliferative activity index; **PI SG (%)**: Sertoli cells proliferative activity index; **GC Desquamation (%)**: Percentage of seminiferous tubule sections containing desquamated cells. **C**: Control in long photoperiod; **MR**: Mild regression; **SR**: Strong regression; **TR**: Total regression. Notably, multiple cellular phenomena occur in MR that together facilitate the initial process of regression of the seminiferous epithelium. These include a decrease in the proliferation of spermatogonia, an increased apoptosis in all germ and Sertoli cells, the appearance of a strong phagocytosis of spermatids, and even a possible increase that is not significant in the preliminary study of germ cell desquamation. Data are obtained from the following references: [15,41,63,64].

An important aspect of this cellular process is whether germ cells undergo an increase in their apoptotic activity. In hare, apoptosis can affect germ cells differently, the phenomenon occurring mainly in spermatocytes and rarely in spermatogonia [52]. In the case of the white-footed mouse or in birds, such as starling [59] and crow [60], apoptosis occurs, not only in spermatocytes, but also in spermatids and, to a lesser extent, in spermatogonia. In the Syrian hamster, spermatocyte apoptosis is known to be essential to maintain testicular regression once it has been established [61,62]. Furthermore, it has been possible to observe and quantify that spermatogonia, spermatocytes, and round spermatids undergo this phenomenon during testicular regression [15]. It is significant that round spermatid apoptosis is a very rare event in testes subjected to a long photoperiod, while it gradually increases during regression. It is also important to highlight the increase in apoptotic activity in spermatogonia during the onset of regression, which is higher than observed in spermatocytes and spermatids before dropping during regression to values similar to those of animals in long photoperiod. Moreover, an unusual activity of the Sertoli cell is the phagocytosis of elongated spermatids during regression of the seminiferous epithelium [63], which aids in its atrophy (Figure 2).

**Figure 3:**
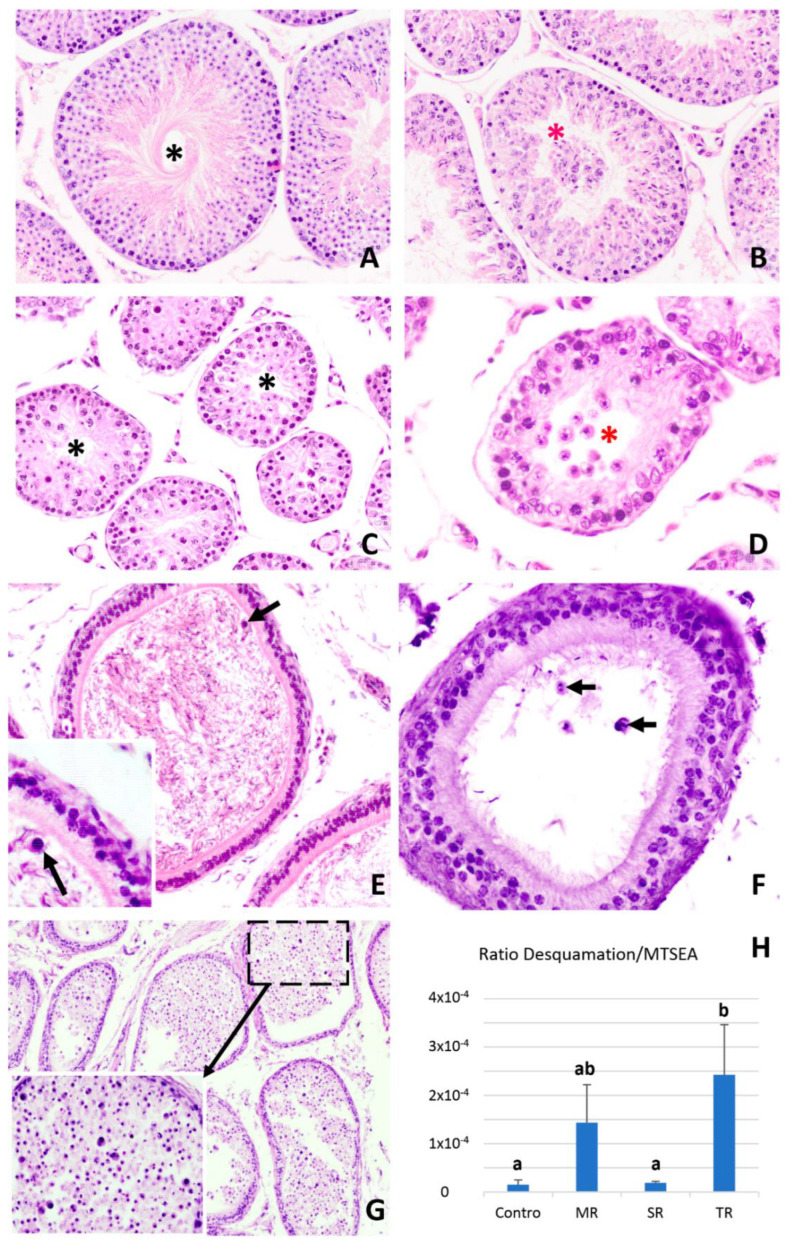
Preliminary results for cell desquamation inside the seminiferous tubule during testicular regression in Syrian hamsters. (**A**–**D**) Tubular section in control (**A**), mild regression (**B**), strong regression (**C**) and total regression (**D**) groups. (**E**–**G**) Cauda of the epididymis in the mild regression (**E**), strong regression (**F**) and total regression (**G**) groups. Black asterisks (**A**,**C**) indicate empty cells in the lumen; red asterisks (**B**,**D**) indicate presence of cells in lumen; black arrows indicate cells in the cauda lumen of the epididymis. (**H**) Preliminary calculation of the ratio between the mean number of desquamated cells (number of spermatocytes + spermatids in the tubular section lumen) in the seminiferous tubules in each study group [67], in relation to the mean tubular seminiferous epithelium area (MTSEA) in each study group [15]. A trend is again observed which could be confirmed in the MR group.

The continued decrease in germ cell numbers in the seminiferous epithelium in the Syrian hamster during testicular regression causes a decrease in the volume and length of the seminiferous tubule. As expected, the epithelial volume also decreases. These phenomena indicate that at the onset of testicular regression the volume of the tubule decreases as its diameter becomes smaller, while the subsequent decrease in tubular volume is caused by the shortening of the tubule itself [15]. This shortening is accompanied by a decrease in interstitial volume (more specifically, the intertubular volume since the peritubular volume does not change). Regarding the Leydig cells located in the testicular interstitium, these decrease in number as testicular regression progresses. This decrease is not due to the decreased proliferation or increased apoptosis of Leydig cells. Rather, an increase in the number of degenerated Leydig cells is observed, showing that the reduction in the number of Leydig cells during testicular regression must be the result of necrosis and/or necroptosis [65]. During the non-breeding season of pheasant, there is a decrease in the interstitial volume as well as in the number of Leydig cells (four times less) [66]. By contrast, in the Iberian mole, no decrease in the number of Leydig cells is observed in the interstitium during testicular regression, when they occupy the entire interstitium after the tubular volume has decreased, which is somewhat similar to what occurs in the roe deer [42]. Finally, in an initial study comparing three regression stages (mild regression (MR), strong regression (SR), and total regression (TR)), we have also observed that germ cell desquamation occurs at the end of the testicular regression process, probably acting as an additional factor in the maintenance of epithelium depletion when fully regressed [67], although it could also be a factor that intervenes at the onset of regression, together with decreased proliferation, increased phagocytosis, and increased germ cell apoptosis (Figure 3).

Similarly, and probably associated with both the reduction in length and volume of the seminiferous tubule and the loss of Leydig cells during regression, an increase in the proliferation of myoid cells and pericytes was observed at the end of regression, probably related to the subsequent recrudescence phase [65,68].

In the recrudescence phase of Syrian hamsters, there is a gradual recovery of the testes, which, at first is related to an increase in tubular length and diameter, as well as interstitial volume, which then depends mainly on the gradual increase in the tubular seminiferous diameter. Recovery of the seminiferous epithelium was accompanied by changes in apoptosis and proliferation activities. The apoptosis decreased halfway through the process, and the proliferation remained higher than the levels of control throughout the recrudescence stage. The final restoration of the seminiferous epithelium is due to the fact that apoptosis reached normal values, which is accompanied by higher proliferative activity than observed in the group of animals with a long photoperiod [69]. As mentioned above, strong immunoreactivity to c-kit was observed in spermatogonia in this initial process, as well as in precursor Leydig cells. The expression of c-kit receptor became weaker as recrudescence progressed and the differentiation of new Leydig cells originating from perivascular cells and peritubular cells was observed [44]. This decrease in c-kit receptor expression during recrudescence may reflect the end of the testicular restoration process. Along with recrudescence, the increase in proliferative activity of myoid cells at the start of this process probably contributes to the recovery of their population, which would explain the significant increase in tubular volume and length during recrudescence [68].

Another species, in which proliferation and apoptosis were observed as important phenomena, as well as involved in the regression of the seminiferous epithelium, is the Daurian ground squirrel (*Spermophilus dauricus*) during the breeding season (April), the non-breeding season (June), and before hibernation on (September). Epithelial regression was recorded during the transition from the breeding to the non-breeding season. Moreover, in June and September, a significant increase in the expression of proliferating cell nuclear antigen (PCNA), cyclin D2, and caspase-3 protein was observed in the testis. Apoptotic cells were found in testicular germ cells only during the prehibernation period. The presence of cyclin D2 in spermatocyte nuclei occurred only in September. This study showed that both cell proliferation and apoptosis increase in the prehibernation period, suggesting they both play an important role in preparing for testicular recrudescence in this species [70].

Several studies have attempted to explain the molecular mechanism that regulates testicular regression in animals, during which proliferation and apoptosis participate in the atrophy of the seminiferous epithelium. Specifically, the increase in apoptosis during regression in the epithelium would be mediated by the increase in the expression of Fas (extrinsic pathway of apoptosis) [49], although in crows, a decrease in the expression of the antiapoptotic factor Bcl-x(L) in Sertoli cells would increase apoptosis in the seminiferous epithelium [60]. In the Japanese quail (*Coturnix japonica*), P53-mediated apoptosis has been observed as responsible for testicular regression [71]. In hamster Fas, Bcl-x (L) and p53 participate in germ cell apoptosis regulation in totally regressed testes (intrinsic and extrinsic pathways of apoptosis), whereas only Bcl-x(L) is involved in the apoptosis associated with the aging of the seminiferous epithelium [72]. Therefore, further studies into molecular pathways are required to determine the role of these molecules during testicular recrudescence.

Taking into account that the currently available data suggest that germ cell apoptosis is the main testis regression effector in birds, reptiles, and amphibians [59,60,71,73,74,75,76], it can be surmised that changes in proliferation and apoptosis are involved in the process of testicular regression in many seasonally breeding vertebrate species. These facts, as mentioned above, have led to the proposal that two tissue models of testicular regression exist, in accordance with whether the apoptosis of Sertoli cells occurs (birds) or not (mammals) [22]. These two models have been lent weight in recent years in the case of birds, in which the death of Sertoli cells has also been observed in other species of birds, such as the crow [60,76]. However, alongside this, evidence has emerged that Sertoli cell death could also be involved in seasonal reproduction-related regression of the seminiferous epithelium in mammals. The first evidence was the observation of a quantitative decrease in Sertoli cells in the fully regressed testes of Russian hamster [77], which seemed to contradict the previous observation in Syrian hamster testes, in which the number of Sertoli cells did not change in fully regressed animals [78]. A second fact was the observation and quantification of Sertoli cell death during the regression of the Syrian hamster [63], raising serious doubts concerning the cellular model proposed by Young et al. (2002) [22].

### 2.3. Proliferation and Apoptosis of Sertoli Cells in the Regression of the Seminiferous Epithelium of Mammals Related to Seasonal Reproduction

Traditionally, it has been considered that, once adult life is reached, the Sertoli cell in mammals is totally differentiated and, as a consequence, quiescent. This assumes that the total number of Sertoli cells is constant and does not change during the individual’s lifetime, when the cells no longer respond to the hormones that previously stimulated them to divide [34,35,36]. However, this view is currently under discussion. Some authors have suggested that the Sertoli cells of adult males can change from their mature and differentiated state to a more immature and undifferentiated state through the effect of gonadotropins, suggesting the existence of a population of Sertoli cells that would be in a dynamic state modifiable by hormones, at least in seasonal breeders, such as the Russian hamster [79]. Recently, our research team has provided new evidence in this regard. Therefore, in the Syrian hamster, we determined that the spontaneous testicular recrudescence after exposure to a short photoperiod leads to an increase in Sertoli proliferative activity to restore the size of the normal population that had been lost during regression. In this species, the increase in Sertoli proliferation from final regression and recrudescence, accompanied by a similar rate of apoptosis as that seen in a control group, is the cause of the restoration of the Sertoli population during spontaneous recrudescence [41].

For many years, it was claimed that apoptosis took place in the Sertoli cells at certain times of the male’s life. Therefore, their apoptosis had only been identified during fetal, prenatal or prepubertal life, while in adulthood, the already quiescent cell underwent an aging process and their numbers did not change [29]. Furthermore, in the last two decades, Sertoli cell apoptosis has been associated with a number of pathologies or the administration of drugs or toxins [80,81]. Under normal physiological conditions, during the regression of the Russian hamster due to a short photoperiod, it has been suggested that the decrease in the population of Sertoli cells could be the result of apoptosis [77]. Moreover, our group has been able to show that during testicular regression in the Syrian hamster, Sertoli cell apoptosis increases, while their total number decreases by more than two thirds of the usual population in the testes subjected to a long photoperiod. In particular, the loss of Sertoli cells occurs at the onset of regression and, once regression is established, apoptotic activity similar to the Sertoli cells during the long photoperiod occurs, but with an increase in proliferative activity [41]. To recapitulate, in the testicular regression of the Syrian hamster, there is an initial phase with decreased proliferation and increased apoptosis in both germ and Sertoli cells. At the same time, strong phagocytosis of spermatids and perhaps the desquamation of cells also occur. Then, the proliferation returns to normal levels in spermatogonia and the apoptotic activity of all germ cells is further increased during mild regression. When complete regression of the seminiferous epithelium is reached, the proliferative activity of Sertoli cells and spermatogonia increases, while apoptosis remains high only in spermatocytes and in the few spermatids that remain. Apoptotic activity in both spermatogonia and Sertoli cells returns to values similar to those of animals in a long photoperiod to prepare for recrudescence. These changes allow us to better understand the reduction in size undergone by the seminiferous tubules, as well as the induction of germ cells to initiate their apoptosis. At the onset of regression, there is a great loss of Sertoli cells, which greatly increases the ratio between germ cells with respect to Sertoli cells, a fact that, in our opinion leads the epithelium to be unsustainable and generates a significant increase in germ cell apoptosis during the following phase of regression [41].

### 2.4. Cell Desquamation of Germinal Cells in the Regression of the Seminiferous Epithelium of Mammals Related to Seasonal Reproduction

In addition to the above evidence, some authors have observed that in various mammalian species, the process of involution of the seminiferous epithelium is cellularly mediated by the strong desquamation of germ cells (sloughing or exfoliation). This desquamation is modulated by the different distributions of the cell adhesion (junctional) molecules between Sertoli–Sertoli cells and Sertoli–germ cells in the seminiferous epithelium. This regression process is followed by a process of spermatogenesis arrest. Later in the final regression, spermatocyte apoptosis occurs, which is important as a factor to prevent the progression of spermatogenesis before the onset of recrudescence. In the seasonal testicular regression of males of the Egyptian long eared-hedgehog (*Hemiechinus auritus*), non-apoptotic germ cells are massively depleted by desquamation [82]. This phenomenon is concomitant with both a decreased level of serum testosterone and the alteration of cell-adhesion molecules between the germinal and Sertoli cells. Previously, this same research group studied the testicular regression in the Iberian mole, *Talpa occidentalis*, finding that apoptosis mainly affected late zygotene and pachytene cells during the period of sexual inactivity, but no increase in apoptosis was observed during testicular regression, when massive germ cell depletion occurred via desquamation. Spermatogonia cell proliferation appeared to restore the number of spermatogonia lost at the end of the testicular regression process. These authors concluded that mammals do not form a homogeneous group regarding the cellular mechanisms that participate in the involution of epithelium in males of seasonally breeding species [83]. Subsequently, these authors presented evidence concerning the role that cell junctions play in the atrophy of the testis of the Iberian mole (*Talpa occidentalis)*. In this species, BTB is altered during testicular regression due to changes in the distribution of adhesion molecules in the seminiferous epithelium. Therefore, it was observed that there is a decrease in the expression of the mRNAs of *CLDN11* and *GJA1* genes. Moreover, the immunohistochemical study allowed for the observation of the positivity to N-cadherin and E-cadherin, as well as alterations with respect to the testis of animals in the reproductive season. The regression period is mediated by low intra-testicular testosterone levels, while Sertoli cells lose their nursing and supporting function, and the impermeability process of the BTB and desquamation cells is rapid [84].

Subsequently, this research group analyzed the changes in the seminiferous epithelium in species of small mammals from the surroundings of the city of Granada, in the south of the Iberian Peninsula. In two of the species, the Algerian mouse, *Mus spretus*, and the greater white-toothed shrew, *Crocidura russula*, the epithelium does not undergo important cellular changes during the winter, which is the non-breeding season [85]. By contrast, in the other two species, the wood mouse, *Apodemus sylvaticus*, and the Mediterranean pine vole, *Microtus duodecimcostatus*, testis regression occurs (facultatively in the latter species) during the non-reproductive period, which takes place in summer [55,58]. These authors concluded that the phylogeny does not seem to be associated with the existence (or not) of seasonal testis regression. From the study of these species, with their diverse cellular mechanisms, they consider that it would be very difficult to establish a general cellular mechanism to explain testicular regression in mammalian species. In this context, a study of the differences at gene expression level between active and inactive testes in different species should aid in explaining which mechanisms and gene pathways are conserved, and which are species specific [58].

Among the species that present cell desquamation as a cellular mechanism that participates in testicular regression is the large hairy armadillo, *Chaetophractus villosus.* This is a seasonal breeder in which a temporary interruption of spermatogenesis has been identified during the period of mid-May to July (mid to end of autumn), accompanied by very low testosterone levels [16], and whose seminiferous epithelium undergoes rapid regression with massive germ cell loss, leaving the tubules with only Sertoli cells and spermatogonia. The testicular regression starts with post-meiotic germ cells sloughing into the tubule lumen and continues with the death of the remaining spermatocytes. Rather, residual meiotic cells undergo all of the stages of apoptosis and are then eliminated by the Sertoli cells [86]. At the end of testicular regression, only spermatogonia and Sertoli cells persist in the seminiferous epithelium. An important characteristic of this animal is the lack of a pineal gland [16]. Moreover, it shows a 12-fold decrease in testosterone levels in the non-breeding season, which causes the adhesion molecules that exist between the Sertoli and germ cells to be altered and the union between them to decrease. This is accompanied by a reduction in vimentin filaments associated with desmosome-like junctions between Sertoli and germ cells. On the basis of these results, the authors suggest that during the non-breeding season there is a loss of adhesion of germ cells to Sertoli cells, which would cause germ cell detachment at the onset of epithelial regression. Using transmission electron microscopy, it was observed that Sertoli cells subsequently phagocytosed cellular debris from germ cells, particularly when testicular regression was reaching its final stages. These findings are new and provide a new mechanism of regression of the seminiferous epithelium specific to this species. In a subsequent study, the authors provided evidence that the localization and quantity of proteins of adhesion complexes present at the apical ectoplasmic junctions (ES) change during testis regression, probably compromising the integrity of the apical ES. This suggests that cell sloughing is determined by changes in the adhesion complexes between Sertoli cells and spermatids, which are mediated by low intra-testicular testosterone levels. In the active testis, B1-integrin, laminin G3, N-cadherin, B-catenin, P-B-catenin-Tyr654, FAK, P-FAK-Tyr397, SRC, P-SRC-Tyr416 proteins present a spermatogenetic cycle-dependent localization pattern, which is not maintained in regressing testes. In the latter, quantitative variations and changes in the phosphorylation state of protein FAK, SRC, and B-catenin contribute to the disassembly of the N-cadherin–N-cadherin and A6B1-integrin–laminin interlocks, thus promoting the massive release of immature spermatids [87].

A special case is the testicular regression observed in plateau pikas (*Ochotona curzoniae*), which starts in early June, while the male pikas are completely infertile, with a dramatically reduced testis size, in late July. In this species, a decrease in germ cell numbers in the testes was first noted in early June. By late June, only Sertoli cells and a small number of spermatogonia remained. Interestingly, large gonocyte-like germ cells were observed in early July. In late July, the number of gonocyte-like cells per tubule increased significantly, and most of the Sertoli cell nuclei clustered in the center of the seminiferous tubules. The gonocyte-like germ and Sertoli cells started to express AP-2γ and anti-Mullerian hormone (AMH) proteins, which were detected in the germ and Sertoli cells of juvenile pikas, but not in adult testes. Simultaneously, LC3 puncta dramatically increased in the seminiferous tubules of the pika testes during the non-breeding season. The study found that spermatogonia and Sertoli cells in non-breeding adult pikas morphologically resembled those in juvenile pikas and expressed specific markers, indicating that dedifferentiation-like transitions may occur during this process. This suggests that de-differentiation of Sertoli cells and autophagy may be involved in this process as a special adaptation of pikas during the non-breeding season [88]. Finally, in Vizcacha (*Lagostomus maximus*), the existence of an association between apoptosis and autophagy has been suggested, which could modulate the changes in the seminiferous epithelium during the reproductive annual cycle of this species. Therefore, it is important to consider that an imbalance between both processes would have important pathophysiological consequences. The results provided in the above study suggest the possible interaction between apoptosis and autophagy in the active and activating testis (characterized by high metabolic and nutrient requirements), whereby autophagy could promote survival over cell death. In the inactive testis, the absence of autophagic-related proteins might explain the massive loss of germ cells, suggesting that autophagy plays a new and key role in the alterations of the seminiferous epithelium during photoregression [89].

To summarize, based on the available information, it seems that apoptosis is the main testis-regression effector in birds and perhaps in reptiles and amphibians (taxa in which more species need to be studied), but not in mammals, where various cellular factors appear to be involved in seasonal testis regression: Cell desquamation, decreased spermatogonia proliferation, apoptosis, autophagy, spermatophagy by Sertoli cells, etc. More specifically, in each species, there seems to be several cellular mechanisms, some of them apparently mainly responsible for depletion of the seminiferous epithelium (the Syrian hamster is a good example). In this species, epithelial depletion is complex, involving the participation of several cellular mechanisms. Early in the regression of the seminiferous epithelium, there is a decrease in the proliferation of spermatogonia accompanied by the apoptosis of germ and Sertoli cells. In addition, the phagocytosis of spermatids and a probable increase in germinal desquamation occur. At a later time, during mid-regression, the apoptotic activity of the germ cells is the preponderant factor, the decrease in the proliferation of the spermatogonia ceases and the apoptotic loss of Sertoli cells continues. Finally, when the highest degree of regression of the epithelium is reached, this is maintained as inactive through the high apoptotic activity of the spermatocytes, while the desquamation of these germinal cells is probably a contributory factor in maintaining epithelial depletion. Meanwhile, the proliferation of spermatogonia and Sertoli cells increases to prepare for recrudescence.

## 3. Conclusions and Future Directions

Following the review of various cellular changes that occur in the testicular regression of the seminiferous epithelium during the non-breeding period in mammals, several questions are still open for discussion. The most important question relates to why various cellular mechanisms exist to achieve depletion of the epithelium. At present, the reasons as to why different species use one or the other is not clear. Nevertheless, there is a certain agreement that the increase in apoptotic activity after the completion of testicular regression is a very common phenomenon, except for species in which the arrest of spermatogenesis has occurred in the spermatogonia. This cellular process ensures that in many species the seminiferous tubules maintain their regressed status during the non-breeding period. In our opinion, taking a dialectical position concerning apoptosis vs. desquamation should be avoided, while research is undertaken into *a paradigm that integrates all the possible biological alternatives* that occur in different species or in the same species without prior viewpoints. It is not a question of finding an explanation that is based on a single mechanism, but of approaching the various types of existing cellular mechanisms, assessing in each species or group of them, how and when they intervene, and what main or complementary role they play. Ultimately, only after a thorough study of the cellular mechanisms involved in regression of the seminiferous epithelium in many species of mammals with seasonal reproduction and of various zoological orders, will it be possible to reach an adequate conceptualization. This will make it easier to find the ultimate biological answer as to why some species opt for a specific mechanism or set of them or why the same species opt for one or the other in accordance with climatic or food conditions. In this sense, the initiation of proteomic and transcriptomic studies of seasonal breeding can shed light on how, in some circumstances or in others, a specific type of cellular mechanism that seeks infertility is activated only when potential offspring cannot survive. It is important that the studies cover the complete reproductive cycle of the species analyzed by performing morphometric observations of both germ cells and Sertoli or interstitial cells. Finally, it is very important that the same degree of understanding that we have of hamster, for which we have a profound knowledge of the effect of different hormones on germ cells or on those of Sertoli or Leydig cells during the reproductive cycle, is reached in other species. Moreover, this information would help better understand inter- or intra-specific cellular changes and differences in seminiferous epithelium regression of various environment alterations, such as light, food, temperature, etc. In conclusion, there is increasing evidence that there are multiple cellular factors that participate in the involution of the seminiferous epithelium during male seasonal reproduction. Indeed, this should not be regarded as unusual, since living species have a high capacity for adaptation, limited by their genetic condition, but modulated by the environment (temperature, light, food, etc.). This translates into a variety of tissue/cellular changes that satisfy the objective of reproduction in the best conditions, ensuring the survival of progeny, and consequently of the species.

## Data Availability

All the data provided have been extracted from the various articles and abstracts consulted.
